# TicTimer software for measuring tic suppression

**DOI:** 10.12688/f1000research.12327.2

**Published:** 2017-12-22

**Authors:** Jonathan K. Black, Jonathan M. Koller, Kevin J. Black

**Affiliations:** 1 Ira A. Fulton College of Engineering and Technology, Brigham Young University, Provo, Utah, 84602, USA; 2 Department of Psychiatry, Washington University School of Medicine , St. Louis , Missouri, 63110, USA; 3 Department of Radiology, Washington University School of Medicine, St. Louis, Missouri, 63110, USA; 4 Department of Neurology, Washington University School of Medicine, St. Louis, Missouri, 63110, USA; 5 Department of Neuroscience, Washington University School of Medicine, St. Louis, Missouri, 63110, USA

**Keywords:** tic disorders, Tourette syndrome, reward, reinforcement, psychology, software

## Abstract

Woods and Himle developed a standardized tic suppression paradigm (TSP) for the experimental setting, to quantify the effects of intentional tic suppression in Tourette syndrome. The present article describes a Java program that automates record keeping and reward dispensing during the several experimental conditions of the TSP. The software can optionally be connected to a commercial reward token dispenser to further automate reward delivery to the participant. The timing of all tics, 10-second tic-free intervals, and dispensed rewards is recorded in plain text files for later analysis. Expected applications include research on Tourette syndrome and related disorders.

## Introduction

Woods and Himle developed a tic suppression paradigm (TSP) that could be used in the experimental setting to demonstrate and quantify the effects of intentional tic suppression on tic rate in Tourette syndrome (TS) and other tic disorders ^1– 5^. In this paradigm, each participant is observed during several experimental conditions, baseline and differential reinforcement of zero-rate ticcing (DRO), and sometimes also verbal instruction to suppress tics and/or noncontingent reinforcement (NCR).

In the course of conducting a longitudinal study of children with Provisional Tic Disorder ^6^, we found that tic suppression is seen within the first few months after a child’s first tic ^7^. We also found that the TSP required substantial investigator effort, and we started writing software with the following goals:

- automated tic counting, timing and record-keeping;
- automated reward delivery in the DRO condition;
- automated reward delivery in the NCR condition.

The overall motivations included not only convenience but also improvement in accuracy. Note that “automated tic counting” here refers to minimizing an expert observer’s record-keeping (simply pushing a button for each tic observed), not to machine detection of the tics. This software, and optional connection to hardware, were intended for use in the research session with the TSP as described above; later in Conclusions we describe possible adaptations for other settings. We present the software here to facilitate its use by others.

## Methods

### Implementation

TicTimer first has the user set up the details for a session, then it runs a clock for the specified session time while writing significant events to a log file. The program writes a line to the log file for each of the following events: session started and ended, tic detected, ten seconds passed without tics, and reward dispensed. Each line includes the time of the event. By parsing through each line in a log file, a python script (also available on GitHub) can extract and summarize the key data.

**Figure 1.**
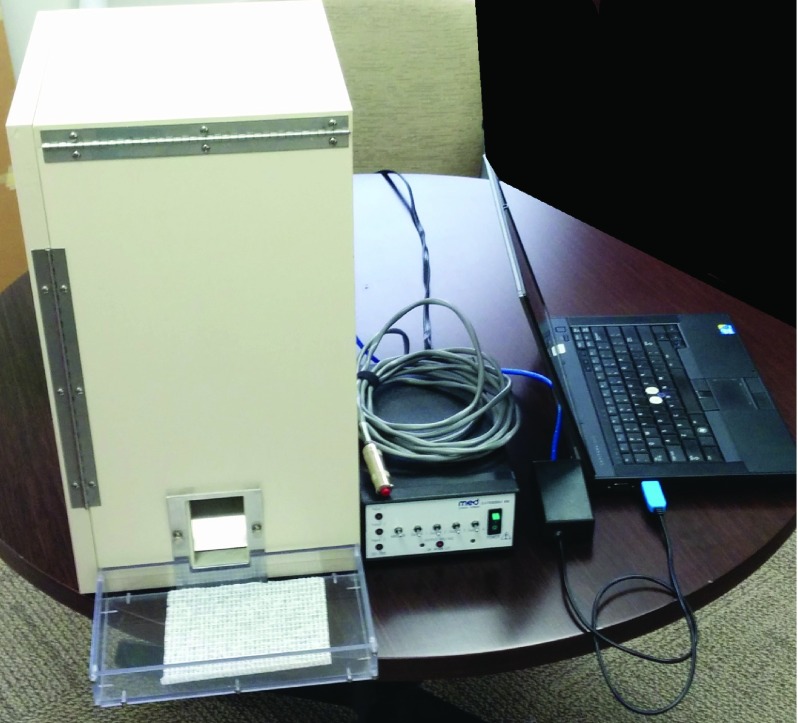
From left to right: Token dispenser box, Student Trainer Interface with external pushbutton, box containing relay module, connected via USB cable to laptop running TicTrainer.

The hardware allows reward tokens to be dispensed automatically to the study participant. One end of the long cable enters the token dispenser and its two wires attach to the two pins in the Passive Connection Panel that, when shorted, trigger release of a reward token. The Student Trainer Interface box provides power to the token dispenser box and a remote pushbutton for manually triggering token release. The other end of the long cable connects to the two normally open pins on the relay inside of the small plastic box. The USB to TTL serial cable attaches to the input pins on the relay, with the USB end of the cable leaving the box to attach to the computer running TicTimer. Figure 1 shows the final assembly.

Parts list:

- Med Associates token dispenser box, Part #ENV-703
- Med Associates SG-595 Student Trainer Interface
- Med Associates SG-215D3 Passive Connection Panel
- 5V Relay module (SainSmart part # 20-018-100)
- USB to TTL serial cable (Adafruit part # 954)
- Plastic project enclosure box (Velleman part # WCAH2855)
- ~6m cable with at least one pair of wires free

### Operation

System software requirements are Java 8 and RXTX for Java, a library for serial port communication. Binaries for Windows and Linux are provided by fizzed.com. Python 3 was used for the log file reader script.

The program can be run with or without the relay and USB cable. If the hardware is set up and connected to the computer, the program can start in “link mode.” Otherwise, TicTimer can still be run in “button mode,” in which the automatic reward system is replaced by a human who presses the push button attached to the Student Trainer Interface when prompted by a beep and a red flash on the computer screen.

The following procedure applies to both reward modes. First, the user presses “Setup” to choose which type of session is being run and to specify where the session log should be saved. For the NCR condition, the user is also prompted to identify the log file from a previously completed DRO session in the same subject (which provides the timing for the rewards dispensed in the NCR condition). Once setup is completed, the session can be started. During a session, the person observing the subject records tics by clicking the “Tic Detected” button or by pressing “T” or the space bar. If the session type includes rewards (DRO and NCR), they are dispensed appropriately. The session ends when the predetermined time elapses or when the user ends it manually by pressing “End Session” or by closing the window.

If a session is ended manually and restarted, the new session log will be appended to the old one unless a new file was chosen in setup. If a log file contains multiple sessions, only the last session will be used by the NCR mode (which requires a DRO session file in setup) and the data reader script.

To summarize the data from a TicTimer log file, run the accompanying python script (TT_Data.py) with one or more log files as arguments. For each log file given, the script reports the length of the session and the number of tics, 10-second tic-free intervals, and rewards dispensed during the session. The choice of 10 seconds as the duration of rewarded tic-free intervals was made to replicate Woods and Himle’s (2004) original methods, and because we have used that duration in all of our own studies.

## Use cases

We have provided 8 sample session log files as Supplementary Files ( Supplementary files 1– Supplementary file 8) ( subject100_session*_TicTimer_log.txt). These are examples of the files that TicTimer creates during a session. Each line in a log file contains an event and the time at which it occurred. Log files are written in plain English, so they can be read directly if desired. These sample data originated from a participant in the study described by Greene *et al.* ^7^, but all identifying data were removed and these files no longer comprise human subjects data.

The file TT_Data_output.txt ( Supplementary file 9) contains output from the python script, summarizing those session files. This output is again in plain text, reporting the session length and the number of each type of event recorded for each log file.

## Conclusions

The TicTimer program, now connected to the reward token dispenser, has simplified implementing the TSP and improved the accuracy of reward delivery (given inevitable limitations of human attention and response time in button mode). The software, while designed for our purposes in tic disorder research, may find other uses. The most obvious of these may be for research on traditional habit disorders; for instance, hair pulling and skin picking appear in the “Obsessive-compulsive and related disorders” section of DSM-5 ^8^. The most obvious application to the clinical setting may be in documenting suppression ability in the office, before and after treatment. However, we have created loosely related web-based software designed for a potential clinical application ^10^. Another potential future modification would be to add machine detection of tics, e.g. by online video analysis or accelerometry; such an improvement would be quite welcome but is difficult to reduce to practice.

## Software availability

The source code is available on GitHub under a BSD 3-clause license.

The current release is available on Zenodo, at DOI 10.5281/zenodo.837884.

## Supplementary material

The first 8 supplementary files are the output from running 8 TicTimer sessions in one subject.

Supplementary File 1: Baseline Log 1

This file came from the first baseline session.

Supplementary File 2: Verbal Log 1

Output from the first session with only verbal instruction to resist tics.

Supplementary File 3: DRO Log 1

Output from the first DRO session.

Supplementary File 4: NCR Log 1

Output from the first NCR session.

Supplementary File 5: Verbal Log 2

Output from the second session with only verbal instruction to resist tics.

Supplementary File 6: Baseline Log 2

Output from the second baseline session.

Supplementary File 7: DRO Log 2

Output from the second DRO session.

Supplementary File 8: NCR Log 2

Output from the second NCR session.

Supplementary File 9: Data Reader Script Output

Output from the command "python TT_Data.py *_log.txt" in the directory containing the 8 sample log files. It summarizes the data contained in those files.
