# Supplementary material for: TicTimer software for measuring tic suppression
Source: F1000Res. 2017 Dec 22;6:1560. Originally published 2017 Aug 24. [Version 2] doi: 10.12688/f1000research.12327.2 (PMC5770993; doi:10.12688/f1000research.12327.2)
Supplement: Peer review history

# Peer review: Referee response for version 2

**DOI:** 10.5256/f1000research.14702.r29268

**Published:** 2018-01-12

**Authors:** Danielle C. Cath

**Affiliations:** 1 Department of Psychiatry, University Medical Centre Groningen, Groningen, Netherlands

I went through the revision and responses by the authors to my comments, and I have no further worries or comments, this work is worthwhile indexing.

I have read this submission. I believe that I have an appropriate level of expertise to confirm that it is of an acceptable scientific standard.

# Peer review: Referee response for version 1

**DOI:** 10.5256/f1000research.13345.r26009

**Published:** 2017-11-06

**Authors:** Patrick Haggard

**Affiliations:** 1 Institute of Cognitive Neuroscience & Department of Psychology, University College London, London, WC1N 3AR, UK

This is a simple but functional piece of software for logging tics, and automating a reward schedule based on them.

 The hardware/software combination will be useful for researchers and possibly for behavioural analysis/clinical training – though this publication does not aim to describe clinical outcomes.

 The software does not appear to actually detect tics automatically – there is no interface to accelometry for example. Rather an examiner seems to have to observe the examinee constantly, and enter manually when a tic occurs, presumably by pressing a button. However, this requirement is unfortunately not specified directly in the text. It also means there is a subjective component: how does the examiner judge what is a tic and what is, for example, a voluntary action: these are tricky questions which face researchers in this area.

 The data is logged in a simple way that is clearly explained, and suitable for postprocessing using standard packages – but the user seems to have to write their own scripts to produce synthetic reports.

 The package logs occurrence of a 10 s period in which no tic is reported – but this time window appears arbitrary: it would be useful (a) to justify it, and (b) to allow the user to change it, either at run time, or in subsequent sensitivity analysis of the log files: how long a period of tic-free behaviour should count as a success event?

 The strength of the tool seems to be the automation of reward scheduling for studies of voluntary tic suppression in children.

I have read this submission. I believe that I have an appropriate level of expertise to confirm that it is of an acceptable scientific standard.

# Response

**Published:** 2017-12-16

**Authors:** Kevin J Black

**Affiliations:** Department of Psychiatry, Washington University in St Louis, USA

We are grateful for the careful attention and thoughtful suggestions of Prof. Haggard. Here we reply to each point in his review of version 1.

- *The software doesn't detect tics automatically; the accuracy of any rater-identified tics depend on the skill of the rater.* These points are all valid. The scope of the software is limited, primarily being useful to track and time the tics as noted by an expert observer, who indeed logs each tic by pressing a keyboard button or clicking a mouse. We have updated the text to clarify these details.
- *"The user seems to have to write their own scripts to produce synthetic reports."* We do supply (via GitHub) the python script TT_Data.py for summarizing each primary data file, but apparently we failed to provide a direct link to this script. We have corrected this omission in this revision.
- *The choice of 10 seconds as the tic-free interval of interest is arbitrary.* We chose the 10s period because of its prior use in the studies cited, but you’re correct that this choice is otherwise arbitrary. We have explained this choice in the revised ms. The suggestions in (b) are interesting, but to some extent outside of our plan for the software. We have modified the source code, however, to make a change in tic-free intervals easier to make at compile time.
- *"The strength of the tool seems to be the automation of reward scheduling for studies of voluntary tic suppression in children.* *"* Yes, we also see that as one of its key advantages. We expect TicTimer could be applied equally well to studies of adults.

# Peer review: Referee response for version 1

**DOI:** 10.5256/f1000research.13345.r27424

**Published:** 2017-10-30

**Authors:** Danielle C. Cath

**Affiliations:** 1 Department of Psychiatry, University Medical Centre Groningen, Groningen, Netherlands

This tool might be very useful in clinical practice to make tic suppression more objectively measured, and to substantiate the direct results of tic suppression practicing. It is potentially a useful addition to current practice of CBT in tic suppression, and entails a worthwhile initiative to develop this tool.

 However, I miss information on some aspects:

1. Direct comparison with other potential ways to generate objective quantifiable results; for instance the advantages compared to video-based tic counting.
2. I miss reflection on potential disadvantages of the tool, and limitations of the tool (for instance in practicing in daily life, not only in the lab, is unclear.
3. In the plain text output, I miss summary information across sessions, and f.i. graphs on tic course across sessions that could be presented to the patient and discussed. The latter would be really helpful for patients and therapist
4. The device can in no way be used in more naturalistic sessions outside the treatment room, the authors should speculate about this, and about future developments to make the tool more practical and applicable in different situations and contexts.
5. I miss pilot information on direct comparisons between conventional tic counting and the counting with the aid of the tictimer.

I have read this submission. I believe that I have an appropriate level of expertise to confirm that it is of an acceptable scientific standard, however I have significant reservations, as outlined above.

# Response

**Published:** 2017-12-16

**Authors:** Kevin J Black

**Affiliations:** Department of Psychiatry, Washington University in St Louis, USA

We thank Prof. Cath for her thoughtful comments. We failed to clarify adequately the primary purpose of this software tool. It was designed rather narrowly for use in a standardized Tic Suppression Protocol in a clinical research office setting. This fact affects many of the specific comments below. We have revised the Introduction to address this point.

Here are responses to each item in your review:

1. *comparing to other tic counting methods:*  This tool can be used (and we have used it) to facilitate counting tics on video recordings. The main drawback with using the software to count and time tics on video recordings is that one cannot give “live” feedback in the Differential Reinforcement of Other (zero-rate ticcing) condition.
2. *limitations:*  We have rewritten the introduction to clarify the limited intended purpose, and clarify in Discussion that use outside of the research office will require adaptations.
3. *graphs showing changes over time, for clinical use:* This is a useful feature that may be added in the future. In the meantime, we point out that the information in Supplementary File 9 can be imported easily into a spreadsheet program for creating such graphs.
4. *can't be used in more naturalistic settings:* Correct. See our initial comment, above. We have in fact adapted some features of TicTimer for a web-based tool intended for more naturalistic (home-based) tic suppression timing. ^[1]^
5. *comparison of TicTimer to conventional tic counting:* We do not feel this is necessary, because (as we apparently failed to explain adequately) in each case an expert human is watching the person with tics. TicTimer adds the (more or less) precise timing of each tic to the record.

[1] Black JK and Black KJ. Software for web-based tic suppression training [version 1; referees: awaiting peer review]. *F1000Research* 2017, **6**:2150 (doi: 10.12688/f1000research.13460.1)
